# Smell loss is associated with cognitive impairment in amyotrophic lateral sclerosis patients

**DOI:** 10.1111/cns.14851

**Published:** 2024-07-08

**Authors:** Xin Huang, Jieying Wu, Nan Zhang, Jinghong Teng, Qiong Yang, Yingshuang Zhang, Tielun Yin, Wen Zhou, Dongsheng Fan, Shan Ye

**Affiliations:** ^1^ Department of Neurology Peking University Third Hospital Beijing China; ^2^ Beijing Key Laboratory of Biomarker and Translational Research in Neurodegenerative Diseases Beijing China; ^3^ Key Laboratory for Neuroscience, National Health Commission/Ministry of Education Peking University Beijing China; ^4^ State Key Laboratory of Brain and Cognitive Science Chinese Academy of Sciences Beijing China; ^5^ Department of Psychology University of Chinese Academy of Sciences Beijing China; ^6^ Department of Neurology Yan'an Hospital of Traditional Chinese Medicine Yan'an China

**Keywords:** amyotrophic lateral sclerosis, cognitive impairment, frontotemporal lobe, smell loss

## Abstract

**Background:**

Smell loss significantly impacts the quality of life in patients. However, there is limited research on smell loss in individuals with amyotrophic lateral sclerosis (ALS), and the correlation between smell loss and cognitive impairment is unclear. This study aimed to investigate the correlation between smell loss and cognition impairment in ALS patients.

**Methods:**

The study included 216 ALS patients. The Edinburgh Cognitive and Behavioural ALS Screen (ECAS) and smell identification test specifically for the Chinese population (CSIT) were administered to evaluate participants' cognitive and olfactory function, respectively.

**Results:**

After covarying for age, sex, BMI, education level, degree of hunger, dietary bias, eagerness for food, stress, smoking status, alcohol consumption, and upper respiratory tract infection (URTI) or rhinitis, CSIT scores were significantly correlated with ECAS scores (*r* = 0.162, *p* = 0.028), especially the ALS‐specific scores (*r* = 0.158, *p* = 0.031). Even after excluding patients with URTI or rhinitis, the results were similar. CSIT scores were significantly correlated with ECAS scores (*r* = 0.224, *p* = 0.011), especially the ALS‐specific scores (*r* = 0.205, *p* = 0.019).

**Conclusion:**

In patients with ALS, smell loss is significantly correlated with cognitive impairment, particularly frontotemporal dysfunction. Cognitive dysfunction may lead to worse olfactory performance in ALS patients.

## Graphical abstract

The olfactory performance of ALS patients is correlated with cognitive impairment, particularly frontotemporal dysfunction.

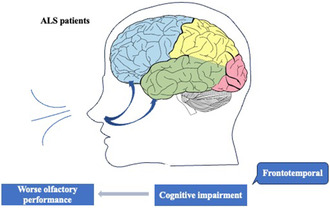

## 1 INTRODUCTION

Amyotrophic lateral sclerosis (ALS) is a devastating neurodegenerative disease characterized by the death of upper and lower motor neurons. Patients with ALS mainly present with limb weakness, dysarthria, dysphagia, and respiratory failure. In addition, a wide range of nonmotor symptoms have been reported, such as cognitive impairment, pain, sleep disruption, autonomic dysfunction, psychiatric disorders, and smell loss.^1^

Smell loss is a common symptom in several neurodegenerative diseases, including Parkinson's disease (PD), Alzheimer's disease (AD), and frontotemporal degeneration (FTD). The loss of the sense of smell has a significant effect on quality of life and thus has received increasing attention in recent years. However, few studies have investigated smell loss in ALS, and their findings are inconsistent. Some studies have reported olfactory loss in patients with ALS,^2^, ^3^ while Lang et al.^4^ found no significant difference in olfactory function between patients with ALS and healthy controls. This inconsistency is thought to be caused by the lack of cognitive examinations.

Cognitive deficits in patients with ALS are common and occur in up to half of these patients.^5^ Currently, ALS and FTD are regarded as a spectrum of diseases.^6^ Studies conducted on FTD and AD patients have revealed a correlation between smell loss and cognitive impairment.^7^, ^8^ Pilotto et al.^9^ demonstrated an association between smell loss and cognitive impairment in ALS‐FTD patients. Future studies focusing on ALS patients with larger sample sizes are needed.

The aim of this study was to explore the correlation between smell loss and cognition impairment in ALS patients.

## 2 MATERIALS AND METHODS

### 2.1 Participants

We screened 245 ALS patients in the Peking University Third Hospital from August 2022 to December 2023. The sample size was decided with reference to other literature. Eventually, 216 ALS patients were included in our study and their baseline characteristics are shown in Table 1. All participants completed the olfactory function test, while 206 ALS patients underwent cognitive assessment. 60 individuals had upper respiratory tract infection (URTI) or rhinitis. This study was approved by the Research Ethics Committee of Peking University Third Hospital. In accordance with the Declaration of Helsinki, written informed consent was obtained from all participants before they were included. The consent procedure was approved by the ethics committee.

**TABLE 1:** Clinical characteristics of amyotrophic lateral sclerosis (ALS) patients.

	ALS (*n* = 216)
Sex, *n* (%), male	136 (63%)
Age, mean (SD), years	50.75 (11.28)
BMI, mean (SD)	23.41 (3.03)
Education level, median (IQR)	12 (9–16)
ECAS score, median (IQR)^a^	100 (82–113.25)
Diagnosis level, *n* (%)	
Definite	60 (27.9%)
Probable	65 (30.2%)
Laboratory‐supported probable	66 (30.7%)
Possible	24 (11.2%)
Diagnostic delay, median (IQR), months	29.43 (14–61.89)
ALSFRS‐R score, median (IQR)	42.5 (39–45)
Degree of hunger, median (IQR)	2 (1–4)
Dietary bias, median (IQR)	2 (1–4)
Eagerness for food, median (IQR)	4 (2–5)
Stress, median (IQR)	4 (2–5)
Smokers, *n* (%)	72 (33.3%)
Alcohol drinkers, *n* (%)	74 (34.3%)
URTI or rhinitis, *n* (%)	60 (27.8%)
CSIT, median (IQR)	30 (26–33)

Abbreviations: ALSFRS‐R, ALS functional rating scale‐revised; BMI, body mass index; ECAS, The Edinburgh Cognitive and Behavioural ALS Screen; IQR, interquartile range; SD, standard deviation.

^a^ 206 of 216 patients had results of ECAS scores.

The inclusion criteria for patients were as follows: (1) diagnosed with clinically definite ALS, clinically probable ALS, clinically probable laboratory‐supported ALS, or clinically possible ALS according to the revised EI Escorial criteria; (2) aged 30–70 years; and (3) voluntarily signed an informed consent (IC) form.

The exclusion criteria for patients were as follows: (1) severe dysarthria or disorders of limb activity, which prevented completion of the neuropsychological examination; (2) nasal or head trauma or a history of nasal surgery; (3) patients who underwent tracheostomy or are using respiratory ventilation; (4) pregnancy; or (5) a history of cognitive disorders or illiteracy.

### 2.2 Neuropsychological assessment

The cognitive function of all the participants was assessed using the Edinburgh Cognitive and Behavioural ALS Screen (ECAS). The ECAS is a cognitive assessment tool designed specifically for ALS patients. The assessment involves an ALS‐specific section (language, verbal fluency, and executive functions, for a total of 100 points) and a non‐ALS‐specific section (memory and visuospatial functions, for a total of 36 points). Higher ECAS total scores indicate better cognitive function.^10^

### 2.3 Olfactory testing

The smell identification test specifically for the Chinese population (CSIT) was developed by the Institute of Psychology, Chinese Academy of Sciences, to evaluate olfactory function.^11^, ^12^ It was adapted from the University of Pennsylvania Smell Identification Test (UPSIT), which is widely used internationally, for use in the Chinese context. In the first part, patient information, including smoking status, alcohol consumption, medical history, and current drug use, was recorded. Whether the subjects had an upper respiratory tract infection (URTI) or rhinitis was also recorded. The second part involves 40 single‐choice questions. All patients and healthy controls were asked to close their eyes and smell an odor (produced by a stimulus placed beneath their nostrils) in a clean, indoor room. Then, they were instructed to identify the odor from four options: coke, ink, garlic, and apple. Their answers were recorded without any feedback. The number of correct answers was summed to calculate the CSIT score. In the last part, the degree of hunger, dietary bias, eagerness for food, and stress were rated by subjects on a scale of 1–7.

### 2.4 Statistical analyses

Shapiro‐Wilk test was performed to determine whether the distribution is normal for all variables. Our study presents continuous variables following a normal distribution as the mean (standard deviation). Non‐normal continuous variables are reported as median (IQR). Categorical variables are expressed as frequencies (percentages). Pearson correlation analysis was used to analyze whether CSIT scores were related to ECAS scores. Then, partial correlation analysis was used to analyze the relationship between CSIT scores and ECAS scores as a multivariable analysis. Confounding factors including age, sex, BMI, education level, degree of hunger, dietary bias, eagerness for food, stress, smoking status, alcohol consumption, and URTI or rhinitis were included in the analysis as covariables. SPSS 27.0 software (IBM Corp., USA) was used to analyze the data. The significance threshold was set at a 2‐tailed *p* < 0.05.

## 3 RESULTS

**FIGURE 1:**
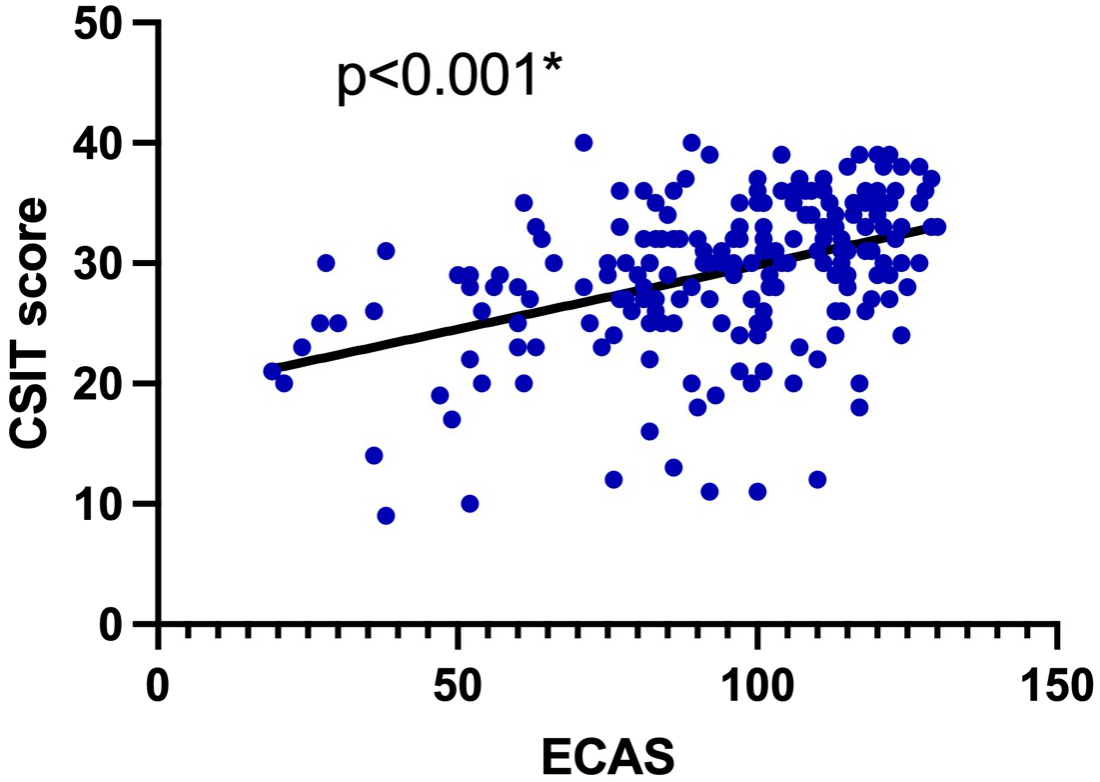
Correlation between CSIT scores and ECAS score in ALS patients. *p < 0.05.

As shown in the scatter plot (Figure 1), there appeared to be a linear correlation between CSIT scores and ECAS scores in ALS patients. In univariable analysis, we used Pearson correlation analysis and found that CSIT scores were significantly related to ECAS scores (*r* = 0.423, *p* < 0.001).

In multivariable analysis, we used partial correlation analysis to investigate their relationship. After adjusting for age, sex, BMI, education level, degree of hunger, dietary bias, eagerness for food, stress, smoking status, alcohol consumption, and URTI or rhinitis, this relationship remained significant between CSIT scores and ECAS scores (*r* = 0.162, *p* = 0.028) (Table 2). Age (*r* = −0.366, *p* < 0.001) and degree of hunger (*r* = −0.147, *p* = 0.045) were also related to CSIT scores. There was no statistically significant relationship between CSIT scores and other factors.

**TABLE 2:** Partial correlation analysis of CSIT scores and other factors.

	ALS patients (overall, *n* = 216)		ALS patients (without URTI or rhinitis, *n* = 156)	
	*r*	*p* Value	*r*	*p* Value
Sex	0.004	0.953	0.038	0.666
Age	−0.366	<0.001*	−0.347	<0.001*
BMI	−0.099	0.180	−0.049	0.582
Education level	0.092	0.214	0.064	0.472
ECAS score	0.162	0.028*	0.224	0.011*
Degree of hunger	−0.147	0.045*	−0.186	0.034*
Dietary bias	−0.098	0.182	−0.073	0.408
Eagerness for food	−0.029	0.691	−0.003	0.970
Stress	−0.084	0.257	−0.122	0.168
Smoking status	−0.106	0.151	−0.067	0.450
Alcohol consumption	0.002	0.978	−0.052	0.556
URTI or rhinitis	−0.047	0.527		

* *p* < 0.05.

URTI or rhinitis might be an important factor influencing olfactory function. We excluded the participants with URTI or rhinitis, which left 156 ALS patients and 75 healthy controls. Similar analyses were performed with data from this subset of participants. The results were similar (Table 2). CSIT scores were correlated with ECAS scores (*r* = 0.224, *p* = 0.011), age (*r* = −0.347, *p* < 0.001), and degree of hunger (*r* = −0.186, *p* = 0.034).

To identify which specific cognitive functions were correlated with olfactory function, we analyzed ALS‐specific and non‐ALS‐specific functions separately. ALS‐specific functions include executive function, language, social cognition, and verbal fluency, which reflect frontotemporal lobe function, while ALS‐nonspecific functions include memory and visuospatial functions. After adjusting for confounding factors including age, sex, BMI, education level, degree of hunger, dietary bias, eagerness for food, stress, smoking status, alcohol consumption, and URTI or rhinitis, partial correlation analyses were conducted to investigate the relationships of CSIT scores with the two ECAS sub‐scores (ALS‐specific and non‐ALS‐specific).

After Bonferroni correction, the relationship between ALS‐specific (*r* = 0.158, *p* = 0.031), non‐ALS‐specific (*r* = 0.071, *p* = 0.335) ECAS scores and CSIT scores did not reach statistical significance (Table 3). However, there may be a trend between olfactory performance and ALS‐specific function. After excluding patients with URTI or rhinitis, the relationship became more significant. CSIT scores were significantly related to ALS‐specific ECAS scores (*r* = 0.205, *p* = 0.019) but not related to non‐ALS‐specific ECAS scores (*r* = 0.127, *p* = 0.150) (Table 3).

**TABLE 3:** Correlations between CSIT scores and ECAS scores (including ALS‐specific and non‐ALS‐specific) in partial correlation analysis.

	ALS patients (overall, *n* = 216)		ALS patients (without URTI or rhinitis, *n* = 156)	
	*r*	*p* Value	*r*	*p* Value
Total score	0.162	0.028*	0.224	0.011*
ALS‐specific score	0.158	0.031	0.205	0.019**
Non‐ALS‐specific score	0.071	0.335	0.127	0.150

*p < 0.05; **p < 0.025.

Additionally, we also compared CSIT scores between ALS patients and healthy controls who were also enrolled during the same period (*n* = 90) in our study. We found that the CSIT scores of ALS patients were significantly lower than controls (*p* < 0.001) (Appendix S1). However, large‐scale studies are necessary furthermore because of the unbalanced baseline characteristics between the two groups in our study.

## 4 DISCUSSION

Our study found that smell loss was correlated with cognitive impairment in ALS patients, especially with ALS‐specific ECAS scores. This result is consistent with previous theories.^13^, ^14^, ^15^, ^16^

Olfaction is a unique sense with its own circuitry. Odors are detected by sensory receptors located in the olfactory epithelium lining the nasal cavity. Sensory neuron axons form the olfactory nerve and carry odor information to the olfactory bulb (OB). The cell axons in the bulb have diffuse projections to multiple cortical regions.^17^ Cortical regions associated with olfaction include the primary olfactory cortex (POC) and secondary olfactory cortex (SOC). The POC is the main cortical region that processes olfactory information and includes the anterior olfactory nucleus (AON) and periamygdaloid complex (PAC)/piriform cortex (PiC). The SOC includes the orbital cortex (OrC) and hippocampal dentate gyrus (DG).^18^, ^19^ After reaching the POC, olfactory signals are further projected to the SOC for integration.^20^

Smell loss is a common symptom in neurodegenerative diseases such as Alzheimer's disease and Parkinson's disease.^21^, ^22^, ^23^ Smell loss was found to be related to the PiC and orbitofrontal cortex (OFC) in PD patients.^24^ In FTD, previous studies have demonstrated that smell loss is associated with the OFC, temporal lobe, and amygdaloid nucleus.^25^

In ALS patients, Verstraete et al.^26^ found that the cortical thicknesses of the precentral gyrus were lower than that of healthy controls. Masuda et al.^27^ found that the odor stick identification test for Japanese (OSIT‐J) was correlated with atrophy of the left medial orbital cortex and right hippocampus in ALS patients through imaging studies. The medial orbital cortex was part of the prefrontal cortex, which played a central role in cognition and behavior. The function of the prefrontal cortex involves attention, execution, decision‐making, and working memory.^28^

The ECAS total score includes both ALS‐specific and non‐ALS‐specific functions. Non‐ALS‐specific functions are common in other neurological disorders and typically remain intact in ALS patients.^29^ ALS‐specific functions are more likely to be affected in ALS patients including language, verbal fluency, and executive functions. Larsson et al.^30^, ^31^ have reported correlations between general semantic memory, verbal fluency, and odor identification in healthy elders. Additionally, Frasnelli et al.^32^ have reported correlations between odor identification ability and gray matter volume from the insular cortex to the superior temporal gyrus.

Our study showed that olfactory function was correlated with cognitive impairment in the ALS population, especially ALS‐specific function which mainly indicated altered function of the frontotemporal lobe.^33^ Thus, we assume that the smell loss in ALS patients might be a result of olfactory recognition and discrimination disorders linked to cognitive impairment. The assumption was supported by previous imaging studies and pathological studies. Pilotto et al.^9^ observed that odor recognition was impaired with normal discrimination in some patients. Such phenomenon was also reported as “odor agnosia” by Takeda et al.^18^ It might be associated with the TDP‐43 pathology of frontotemporal and insular cortical/subcortical areas.^18^, ^34^ However, the effect of olfactory perception dysfunction cannot be totally excluded because the pathology involves peripheral olfactory structures such as OB as well.^35^ We suppose that the central and peripheral part of the olfactory pathway are both impaired. Cognitive deficits due to frontotemporal lobe damage may affect olfactory recognition and discrimination, leading to a diagnosis of smell loss. However, the underlying mechanism remains unclear.

In line with the hypothesis, odor identification deficit was also reported in patients with FTD.^25^, ^36^, ^37^, ^38^ Furthermore, the deficit was more prominent in patients with greater impairment of temporal lobes on MRI.^37^, ^39^ Orasji et al. also reported that odor dysfunction may be due to disrupted areas in the temporal lobe and amygdala.^40^

Compared with previous studies,^26^, ^27^ the present study had a larger ALS cohort and indicated that smell loss in ALS patients might be due to frontotemporal cognitive impairment; indeed, cognitive impairment may be a more important factor than olfactory function (e.g., functioning of sensory receptors) in determining olfactory performance. This finding indicates a new avenue for further research.

There are several limitations of our study. First, our participants were recruited from a single center, which limits the generalizability of our results. Second, the number of healthy controls was small, and the healthy controls were not matched to ALS patients in terms of sex, age, BMI, or education level. Thus, the result of the comparison between ALS patients and healthy controls was not included in the manuscript but provided in the Appendix S1. Third, imaging and biological examinations are needed to validate the results further.

## 5 CONCLUSIONS

Olfactory performance is significantly correlated with cognitive function, especially frontotemporal function, in the ALS population. Cognitive impairments might cause the reduced olfactory performance observed in ALS patients. Further research is needed to explore the underlying mechanism.

## AUTHOR CONTRIBUTIONS

Conceptualization, S.Y.; Methodology, X.H., W.Z., and S.Y; Resources, Q.Y., Y.Z., T.Y., and W.Z.; Data Curation, X.H., J.W., N.Z., and J.T.; Writing—Original Draft Preparation, X.H.; Writing—Review and Editing, S.Y.; and Supervision and Project Administration, D.F.

## FUNDING INFORMATION

This work was supported by grants from the National Natural Science Foundation of China (82001350), Beijing E‐Town Cooperation & Development Foundation (YJXJ‐JZ‐2021‐0014 and YCXJ‐JZ‐2022‐007), PUTH Cohort Construction Project (DL2019002), STI2030‐Major Projects (2021ZD0204200), and The Chinese Academy of Sciences Grants (JCTD‐2021‐06).

## CONFLICT OF INTEREST STATEMENT

The authors declare no conflict of interest.

## INFORMED CONSENT STATEMENT

Informed consent was obtained from all subjects involved in the study.

## Supporting information


Appendix S1.

## Data Availability

The data that support the findings of this study are available from the corresponding author upon reasonable request.
