# Supplementary material for: Smell loss is associated with cognitive impairment in amyotrophic lateral sclerosis patients
Source: CNS Neurosci Ther. 2024 Jul 8;30(7):e14851. doi: 10.1111/cns.14851 (PMC11230928; doi:10.1111/cns.14851)

**Supplementary Content**

Manuscript ID: CNSNT-2024-614

Manuscript Title: Smell Loss is associated with Cognitive Impairment in Amyotrophic Lateral Sclerosis Patients

Authors: Xin Huang†, Jieying Wu†, Nan Zhang, Jinghong Teng, Qiong Yang, Yingshuang Zhang, Tielun Yin, Wen Zhou, Dongsheng Fan* and Shan Ye*

Healthy controls were recruited from patient caregivers who had similar living environments. The inclusion criteria for controls were as follows: (1) aged 30-70 years; and (2) voluntarily signed an informed consent (IC) form.

The exclusion criteria for controls were as follows: (1) a history of central nervous system diseases, such as stroke, cognitive disorders, PD, or ALS; (2) nasal or head trauma or a history of nasal surgery; (3) a history of psychiatric disorders; (4) pregnancy; or (5) a history of cognitive disorders or illiteracy.

**Supplementary Table**. Clinical characteristics of healthy controls(n=90) and comparison between ALS patients and controls.

|  | **Controls(n=90)** | **P-value** |
| --- | --- | --- |
| ﻿Sex, n (%), male | 30 (33.3%) | <0.001* |
| Age, mean (SD), y | 45.16 (12.07) | <0.001* |
| BMI, mean (SD) | 25.17 (4.29) | <0.001* |
| Education level,  median (IQR) | 16 (9-16) ^a^ | 0.161 |
| ECAS score, median (IQR) | 114 (98.25-120.75) ^b^ | <0.001* |
| Degree of hunger,  median (IQR) | 1.5 (1-3) | 0.01* |
| Dietary bias, median (IQR) | 2 (1-3) | 0.158 |
| Eagerness for food,  median (IQR) | 4 (2.75-6) | 0.484 |
| Stress, median (IQR) | 3 (1-4) | 0.053 |
| Smokers, n (%) | 17 (18.9) | 0.009* |
| Alcohol drinkers, n (%) | 20 (22.2%) | 0.028* |
| URTI or rhinitis, n (%) | 15 (16.7%) | 0.031* |
| CSIT, median (IQR) | 34(31-37) | <0.001* |

BMI, body mass index; ECAS, The Edinburgh Cognitive and Behavioural ALS Screen; SD, standard deviation; IQR, interquartile range.

The comparison between ALS patients and controls was performed using Student’s t test (for normal continuous data), the Mann‒Whitney U test (for non-normal continuous data) and the chi-square test (for categorical data).

^a^. 49 of 90 controls provided education levels.

^b^. 48 of 90 controls completed ECAS.

* p<0.05

**Supplementary Figure.** Comparison of the CSIT scores between ALS patients and healthy controls.


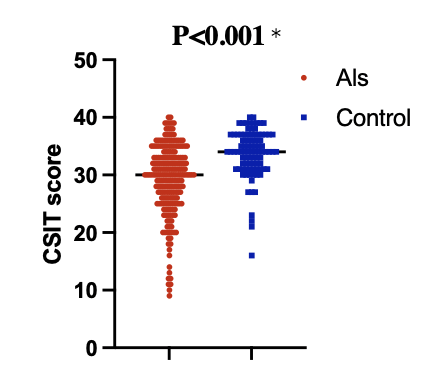

Supplement: Supplementary file 1 — Appendix S1. [file CNS-30-e14851-s001.docx]
